# What are colorectal cancer survivors’ preferences for dietary advice? A best-worst discrete choice experiment

**DOI:** 10.1007/s11764-017-0615-2

**Published:** 2017-04-20

**Authors:** Stuart J. Wright, Debbie Gibson, Martin Eden, Simon Lal, Chris Todd, Andy Ness, Sorrel Burden

**Affiliations:** 1 Manchester Centre for Health Economics, The University of Manchester, Manchester, M13 9PL UK; 2 Manchester Academic Health Sciences Centre (MAHSC), Manchester, M13 9NT UK; 3 School of Health Sciences, The University of Manchester, Jean McFarlane Building, Oxford Road, Manchester, M13 9PL UK; 4 School of Medical Science, The University of Manchester, Manchester, M13 9PL UK; 5 Salford Royal NHS Foundation Trust, Salford, M6 8HD UK; 6 The NIHR Biomedical Research Unit in Nutrition, Diet and Lifestyle, University Hospitals Bristol NHS Foundation Trust and the University of Bristol, Bristol, BS2 8AE UK

**Keywords:** Colorectal cancer, Survivorship, Information dietary, Discrete choice experiments, Conjoint experiments

## Abstract

**Purpose:**

Studies on healthy lifestyle interventions in survivors of colorectal cancer have been disappointing, demonstrating only modest changes. This study aims to quantify people’s preferences for different aspects of dietary intervention.

**Method:**

A best-worst discrete choice experiment was designed and incorporated into a questionnaire including participants’ characteristics and a self-assessment of lifestyle.

**Results:**

The response rate was 68% and 179 questionnaires were analysed. When analysing aggregate preferences, the modes of information provision selected as the most preferred were “face-to-face” (willingness to pay (WTP) £63.97, *p* ≤ 0.001) and “telephone” (WTP £62.36, *p* < 0.001) discussions whereas group discussions were preferred least (WTP −£118.96, *p* ≤ 0.001). Scenarios that included hospitals were most preferred (WTP £17.94, *p* = 0.031), and the favoured provider was bowel cancer nurses (WTP £75.11, *p* ≤ 0.001). When investigating preference heterogeneity, three sub-groups were identified: Firstly, “technophiles” preferring email (WTP £239.60, *p* ≤ 0.001) were male, were younger and had fewer risk factors. Secondly, a “one-to-one” group had strong preference for interventions over the telephone or at their local doctors and were older (WTP £642.13, *p* ≤ 0.001). Finally, a “person-centred” group preferred face-to-face individual or group sessions (WTP £358.79, *p* < 0.001) and had a high risk lifestyle.

**Conclusion:**

For survivors of colorectal cancer, there is not one approach that suits all when it comes to providing dietary advice.

**Implications for Cancer Survivors:**

This is important information to consider when planning healthy lifestyle interventions which include dietary advice for survivors of colorectal cancer. Aligning services to individuals’ preferences has the potential to improve patient experience and outcomes by increasing uptake of healthy lifestyle advice services and promoting a more tailored approach to dietary modifications, acknowledging sub-groups of people within the total population of colorectal cancer survivors.

**Electronic supplementary material:**

The online version of this article (doi:10.1007/s11764-017-0615-2) contains supplementary material, which is available to authorized users.

## Introduction

Colorectal cancer is the third commonest cancer in men and the second commonest in women [1]. Cancer survival rates are continually improving [2]. Consequently, the healthcare needs of people surviving colorectal cancer are becoming increasingly important [3].

People who survive cancer have increased health needs compared to controls [4–6]. In addition to this, “people in survivorship” continue to have higher levels of health risks, including a greater risk of cardiovascular disease, diabetes, secondary malignancies and other cancers [7, 8]. This has been shown to lead to decreased economic productivity resulting from increased levels of overall morbidity and ongoing disability associated with primary malignancy [9]. It is known that people who have survived cancer are motivated to change their lifestyle, and this is termed a “teachable moment” when it is ideal to intervene. [10].

Telephone interventions have been shown to improve healthy eating and physical activity in people with colorectal cancer in relation to exercise and body mass index (BMI), although limited efficacy was demonstrated in terms of improving fruit, fibre and alcohol intakes [11]. Face-to-face counselling combined with telephone support has been shown to reduce body weight and BMI [12]. Whilst benefits from health promotion have been demonstrated in survivorship, results have been limited to relatively modest reductions in body weight and limited changes in dietary intake. Evidence assessing the preferences of people with colorectal cancer to guide service development is currently lacking. Aligning services to people’s preferences to improve uptake is somewhat intuitive but supported by the high dropout rates and the number of participants who decline to take part in clinical trials designed to evaluate a dietary intervention using a single method of delivery [12, 13].

The current evidence base, although demonstrating some positive effects of interventions [14, 15], has not been fully implemented into long-term management or service delivery, possibly due to lack of both cost-effectiveness data or evidence of long-term benefits. Health promotion interventions are recommended as part of colorectal cancer survivorship care, but the specific recommendations on dietary interventions for maintaining or achieving a healthy weight and eating a diet high in fruit and vegetables are derived from evidence based on expert opinion or case studies [16]. Any systematic attempt aimed at changing the lifestyle behaviour of individuals is a complex intervention. Complexity in relation to these interventions manifests in a number of ways including mode of delivery, duration, targeted sample, professional providing the intervention and place. Cognisant of the anticipated challenges, guidance on the development and evaluation of complex interventions has been developed [17].

Identifying patients’ preferences for the design of such an intervention may help to engage service users and to increase uptake. However, it is difficult to observe patients’ preferences for healthcare interventions in practice. Traditionally economic evaluation has relied on revealed preferences, drawing inferences from how individuals act in a market. However, given that healthcare is publicly provided in the UK, such preferences cannot be observed. Discrete choice experiments (DCEs), a type of stated preference study, have been used in health service research to determine which aspects of healthcare delivery are most valued by its users [18]. In a DCE, hypothetical healthcare services are described using a set of pre-defined attributes and participants are asked to choose which of the presented scenarios they would prefer to access.

Design and analysis of DCEs is based on the proposition that people choose goods or services based on their preferences for individual characteristics of the goods [19]. Economic models can be used to quantify the relative strength of preferences which participants have for different aspects of a service based on their choices. There is a synergy between the theoretical basis of DCEs and recommendations for the evaluation of complex interventions. Medical Research Council guidance highlights a need, during development of new interventions, to identify “the active ingredients and how they are exerting their effect” [17].

A key advantage of DCEs is the ability to measure preferences for the outcomes of an intervention and also for how it will be delivered in practice. DCEs have been used for a number of years in a variety of healthcare environments and have now developed methodologically to include best-worst scaling discrete choice experiments (BWDCEs) [20]. In BWDCEs, participants indicate their favourite and least favourite options from a set of three or more scenarios, and BWDCEs elicit more data from participants than a traditional DCE without overburdening respondents [19, 21]. By identifying how different groups of individuals vary in their preferences, services can be further tailored to meet the demands of patients. DCEs therefore have the potential to be an effective tool in addressing two of the key issues in the evaluation of complex interventions: they can assist with issues relating to a lack of effect by evaluating implementation (or uptake) rather than focusing on ineffectiveness and they can also determine if “strict standardisation may be inappropriate”, with adaptation of the interventions which may potentially lead to greater benefits [17].

The aim of this project was to identify and quantify people’s preferences for different aspects of a diet-based lifestyle intervention for people following treatment for colorectal cancer.

## Methods

**Fig. 1:**
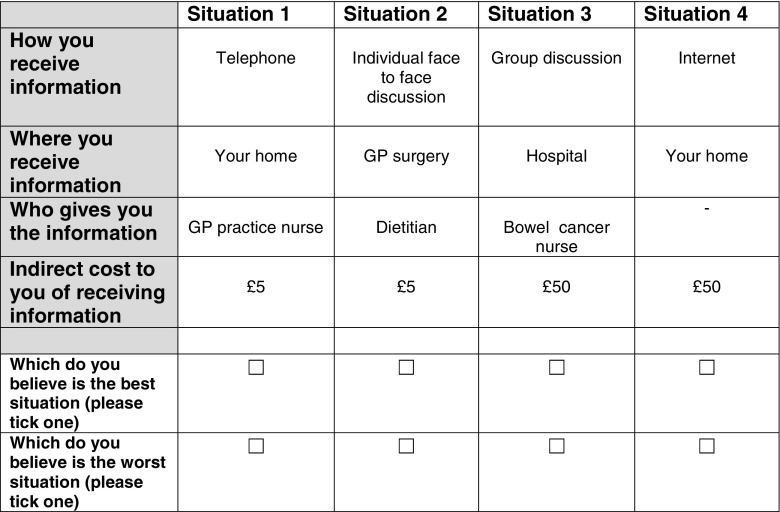
An example question with different attributes and levels

A questionnaire including a BWDCE (Fig. 1) was developed and given to people who were recruited from colorectal follow-up clinics between June 2015 and September 2015. Research nurses recruited participants and provided them with a paper version of the questionnaire and a postage paid envelope to return the questionnaire to the research team. Participants were included if they had completed treatment for colorectal tumour, were over 18 years old, could understand written English and could complete a questionnaire. Those still in receipt of any anticancer therapy were excluded, as were children and those who could not read or write English.

The BWDCE was combined with some questions to collect data on participants’ characteristics including age, gender, occupation, household income, marital status, site of surgery, centre, ethnicity, smoking status, and number of healthy lifestyle criteria that they followed based on international criteria [20]. Participants were asked to indicate their most and least preferred way of receiving dietary information from a range of scenarios with a number of attributes (Fig. 1). These were then ascribed an arbitrary cost that enabled an economic value or willingness to pay (WTP) to be attributed to each best or worst choice.

Attributes were developed from a previous set of qualitative interviews with 32 participants who had survived colorectal cancer [22]. Semi-structured interviews were conducted that asked participants where they received healthy lifestyle information and their preferences about delivery including place, format and personnel giving the information. These were taken as the attributes for the BWDCE, and the levels of each attribute were determined from the range of responses given by the interview participants. This was complemented by a systematic review of intervention trials undertaken with people who had survived cancer [23]. A group of cancer survivors commented on the questionnaire prior to finalising the structure and content.

The questionnaire included scenarios made up of different attributes, and each attribute had a number of levels. The experimental design software program NGene was used to create a *D*-efficient design for the survey [24]. Only one set of 12 choice questions was created as the use of four profiles in each set and the two choices (best and worst) made by each participant was believed to provide sufficient statistical strength to the design without the need for blocking sets of questions. To improve response efficiency to make all profiles realistic, various combinations of levels were prevented from appearing together. For example, telephone calls and emails could only be received at home. An effective experimental design ensures that the coefficients of interest can be precisely estimated.

Participants were asked to complete 12 choice sets in which they had to choose their most and least preferred options from a set of hypothetical diet-based interventions, with each set containing four full profiles of attributes and levels. No opt-out option (for example, receiving no information) was included because of the large number of scenarios to be evaluated. To understand how respondents completed the questionnaire, questions were included to ascertain how difficult participants found the task, whether they used all of the attributes and levels or a sub-section to choose between alternatives, and which attribute or level they found most important to them.

The DCE was initially piloted by 20 participants, and a patient and public engagement group commented on the questionnaire. Participants completed the experiment and then discussed the content. No significant changes to the survey were highlighted by the groups, and pilot but preliminary analysis of the data suggested that too many restrictions to the combinations of attributes and levels had been included. As such, a new *D*-efficient design was created where individuals could receive a discussion in their own home. This allowed parameter estimates for all attributes and levels to be identified in the full analysis.

### Statistical analysis

A minimum sample size of 167 was determined necessary in order to obtain reliable estimates of the coefficients of interest. Details of the sample size calculations for this study can be found in the technical supplementary material. Responses were initially separated into best only and worst only and analysed using conditional logistic regression models in the statistical analysis software Stata 14 [25]. The combined data were then analysed using a sequential best-worst logistic regression in Latent Gold Choice, which better facilitates the analysis of best-worst data and latent choice models [26]. The inclusion of a cost attribute allowed for the calculation of how much participants would be willing to pay for each aspect of the intervention. The WTP values were calculated using coefficients from regression models (see supplementary material for WTP calculations).

Finally, preference heterogeneity was examined using scale-adjusted latent class sequential best-worst logistic regression analysis. This method of data analysis splits the sample up into groups which have similar preferences for the attributes and levels. The demographic information, which was collected alongside the choice data, was then used to identify the characteristics that predicted membership of different categories. A scale-adjusted model [27] was used to account for the fact that participants may have different levels of consistency in their choices, a factor which can confound preference estimates and make them difficult to compare.

## Results

A total of 265 participants were approached, and 190 questionnaires were returned from individuals attending six hospitals throughout England. The study was registered on the Clinical Research Network Portfolio for studies conducted in England; therefore, hospitals were able volunteered to run the survey, and each institution provided a research nurse to support recruitment. During data cleaning, 11 responses were excluded due to missing data, leaving 179 questionnaires for the final analysis (response rate 68%). This relatively high response rate is in line with findings which suggest that patients who have experience of a disease may provide higher response rates to DCE questionnaires [28]. Participants’ characteristics are shown in Table 1; their mean age was 68.9 (SD 8.6). The proportions of male and female participants were similar (51 to 49%, respectively), and most participants were married or living with a partner (66%). In this study, a surprisingly large number of participants had no formal education (36%), which may have implications for the generalisability of the findings to people with different educational levels.

**Table 1:** Characteristics of the respondents

Characteristics	*n*(%)
Gender	
Men	91(51)
Women	88(49)
Marital status	
Single	13(7.2)
Living with partner	11(6.1)
Married	107(60)
Separated/divorced	21(12)
Widowed	27(15)
Stoma	
Yes	44(24)
No	134(75)
Smoking status	
Current smoker	99(55)
Ex-smoker	8(4.4)
Never smoked	72(40)
Income per month	
Under £250	4(2.2)
£250–£500	13(7.2)
£501–£1000	22(12)
£1001–£2000	35(19)
Over £2000	38(21)
Did not answer	67(37)
Ethnic origin	
White British	170(94)
Irish	4(2.2)
Pakistani	1(0.5)
Other	4(2.2)
Cancer site	
Bowel	132(73)
Rectum	23(13)
Bowel and rectum	17(9.4)
Other	7(4)
Education level	
None	65(36)
Trade/NVQ	23(13)
GCSE level	31(17)
A level or equivalent	20(11)
Degree	32(18)
Higher degree	2(1)
Missing	4(2)
Number of participants meeting healthy lifestyle recommendations	
Healthy weight for height	113(63)
Moderate exercise at least 30 min a day	161(90)
5 pieces of fruit and vegetables	104(58)
Not having a lot of sweet food	130(73)
Not having a lot of food high in fat	155(86)
Having red meat <3 times a week	136(76)
Having <2 alcoholic drinks	112(62)

There were 70 (39%) participants who indicated that they found the BWDCE to be easy, and 3 (2%) participants reported finding the study to be very hard. When asked which attributes participants used to make their choices, 143 (80%) stated that they used the “How” attribute, 130 (73%) stated that they used the “Where” attribute, 137 (77%) stated that they used the “Who” attribute and 52 (29%) stated that they used the cost attribute. Respondents could indicate more than one attribute. The modal choice of the most important attribute was “How”, indicated by 77 (43%) participants, whilst “Who” was also perceived to be important, indicated by 64 (36%) participants. Only 2 participants stated that cost was the most important attribute.

### Identification of best and worst aspects of a healthy lifestyle intervention

Conditional logistic regression of profiles which were chosen as the best and worst in each choice task are shown in Table 2. Participants favoured profiles in which information was provided via either telephone calls (*p* < 0.001) or individual face-to-face discussions (*p* < 0.001). They preferred to receive this information in hospitals (*p* = 0.031) and for specialist bowel cancer nurses to provide the information (*p* < 0.001). Group discussions (*p* < 0.001), information provided in community centres (*p* < 0.001) and information provided by general practice nurses (*p* < 0.001) were all levels that were disliked by participants.

**Table 2:** Best and worst case preferences for scenarios showing attributes and levels

Attribute/level	Predictors of best scenarios			Predictors of worst scenarios			Level of agreement
	Coefficient	*p* value	WTP	Coefficient	*p* value	WTP	
Mode							
Telephone	0.515***	0.001	£62.35	0.139	0.153	−£56.87	Somewhat contradict
Discussion	0.528***	<0.001	£63.97	−1.466***	0.001	£599.10	Agree
Group discussion	−0.982***	<0.001	−£118.96	−0.062	0.572	£25.44	Somewhat contradict
Email	−0.061		−£7.39	1.389		−£567.68	Somewhat agree
Location							
Hospital	0.148*	0.031	£17.94	0.372***	0.001	−£152.13	Contradict
GP	0.108	0.073	£13.14	0.088	0.258	−£35.87	Agree (insignificant)
Community centre	−0.220***	<0.001	−£26.70	0.310***	0.001	−£126.78	Agree
Own home	−0.036		−£4.36	−0.770		£314.79	Somewhat contradict
Provider							
Bowel cancer nurse	0.620***	<0.00	£75.11	−0.326***	0.001	£133.32	Agree
Dietician	−0.081*	0.037	−£9.76	0.138***	0.001	−£56.59	Agree
General nurse	−0.539		−£65.31	0.188		−£76.73	Agree
Cost	−0.008***	<0.001		0.002**	0.002		

*WTP* willingness to pay

*p ≤ 0.05, **p ≤ 0.005, ***p ≤ 0.001

Individual discussions were unlikely to be chosen as the worst way of receiving information. This seems congruent with findings of the best case scenario analysis in that this mode of information provision was not associated with an increased likelihood of a profile being chosen as the best. The level of apparent (dis)agreement between the separate analyses of best and worst options is detailed in Table 2. Participants’ disliking of community centres and general practice nurses along with their preference for information from bowel cancer nurses echoes the findings of the best case analysis. However, receiving information in a hospital was found to increase the chance of a profile being selected as worst. This level also tended to appear in profiles which were selected as best, presenting ostensibly conflicting evidence. Other apparent contradictions included preferences for telephone discussions, group discussions and receiving information in a patients’ own home, although in each case, one of the WTP values generated in the best or worst case analysis was statistically insignificant. These examples of counterintuitive findings are indicative of preference heterogeneity in the group.

### Sequential best-worst case analysis

The results of the sequential best-worst logistic regression are presented in Table 3. Telephone calls and individual discussion were the most preferred methods of receiving information with group discussions and emails being disliked. Participants wanted to receive information at their general practice (GP) or in their own home and not in a community centre. The value for information received in a hospital may again indicate varying preferences for a hospital-based intervention. Participants overwhelmingly wanted information from a specialised bowel cancer nurse rather than a dietician or a general practice nurse. Profiles with higher costs were preferred less, as would be expected. WTP values are included to indicate how valuable the different attributes and levels are relative to the mean value of healthy lifestyle and dietary advice.

**Table 3:** Sequential best-worst logistic regression

Attribute/level	Coefficient	*p* value	WTP
Constants			
1	0.0529	0.057	
2	0.0223	0.429	
3	−0.0519	0.062	
4	−0.0233	0.411	
How information is provided			
Telephone	0.2384***	<0.001	£64.62
Discussion	0.7795***	<0.001	£211.27
Group discussion	−0.3186***	<0.001	−£86.36
Email	−0.6993***	<0.001	−£189.54
Where information is provided			
Hospital	−0.0582	0.191	−£15.78
GP	0.1119**	0.004	£30.32
Community centre	−0.2216***	<0.001	−£60.06
Own home	0.168**	0.004	£45.53
Who provides information			
Bowel cancer nurse	0.4641***	<0.001	£125.8
Dietician	−0.1116***	<0.001	−£30.24
General nurse	−0.3526***	<0.001	−£95.57
Cost of receiving information	−0.0037***	<0.001	

*WTP* willingness to pay

*p ≤ 0.050, **p ≤ 0.005, ***p ≤ 0.001

### Determining categories using latent class analysis

A scale-adjusted latent class sequential best-worst logistic regression was used to account for heterogeneity within preferences by identifying groups of participants with similar preferences (see supplementary material for further details).

The results of the scale-adjusted latent class sequential best-worst logistic regression are presented in Table 4. The key difference between the three categories was in their preferences for how they received advice. The first category (*n* = 79, 44%), “technophiles”, preferred indirect communication and was the only group who valued receiving information by email. They were willing to pay an additional £239.60 relative to the average for a dietary intervention. They also valued receiving dietary advice via telephone or in an individual discussion. This group preferred to visit their GP to receive the information or to receive it in their own home and did not want to travel to a hospital or a community centre. Members of the technophiles group were more likely to be younger, to be male and to have low self-reported risk.

**Table 4:** Scale-adjusted latent class sequential best-worst logistic regressionᅟ

	Group 1: “technophiles” (*n* = 44%)			Group 2: “one-to-one” (*n* = 34%)			Group 3: “person-centred” (*n* = 22%)		
Attribute/levels	Coefficient	*p* value	WTP	Coefficient	*p* value	WTP	Coefficient	*p* value	WTP
Constants									
1	−0.010	0.920		−0.036	0.788		0.018	0.913	
2	−0.036	0.737		−0.060	0.665		0.233	0.184	
3	−0.049	0.628		−0.024	0.865		−0.071	0.673	
4	0.095	0.388		0.119	0.418		−0.180	0.313	
How information is provided									
Telephone	1.001***	0.000	£115.54	3.828***	0.000	£642.13	−3.251***	0.000	−£276.78
Individual discussion	0.682***	0.000	£78.77	3.543***	0.000	£594.32	4.214***	0.000	£358.79
Group discussion	−3.758***	0.000	−£433.90	0.680*	0.022	£114.06	3.414***	0.000	£290.62
Email	2.075***	0.000	£239.60	−8.052***	0.000	−£1350.51	−4.377***	0.000	−£372.62
Where information is provided									
Hospital	−0.605***	0.001	−£69.89	−0.484	0.055	−£81.20	0.145	0.567	£12.35
GP surgery	0.638***	0.000	£73.64	0.786***	0.000	£131.81	0.011	0.955	£0.90
Community centre	−0.526***	0.000	−£60.74	−0.798***	0.000	−£133.90	−0.258	0.195	−£21.95
Own home	0.494**	0.009	£56.99	0.497	0.052	£83.29	0.102	0.734	£8.69
Who provides information									
Bowel cancer nurse	1.152***	0.000	£133.02	1.450***	0.000	£243.26	0.986***	0.000	£83.92
Dietician	−0.333***	0.001	−£38.49	−0.776***	0.000	−£130.20	0.080	0.610	£6.84
General nurse	−0.819***	0.000	−£94.53	−0.674***	0.000	−£113.06	−1.066***	0.000	−£90.76
Cost of receiving information	−0.009***	0.001		−0.006*	0.030		−0.012***	0.000	
Class covariates									
Bowel cancer recommendation score	0.295**	0.002		0.020	0.833		−0.315**	0.003	
Age	−0.043***	0.000		0.024*	0.043		0.020	0.138	
Male	0.370**	0.002		−0.180	0.129		−0.190***	0.000	
Female	−0.370**	0.002		0.180	0.129		0.190***	0.000	

*WTP* willingness to pay

*p ≤ 0.05, **p ≤ 0a.005, ***p ≤ 0.001

The second category (*n* = 61, 34%) had preferences indicating they preferred “one-to-one” communication. This was exhibited in this group’s strong preferences for receiving information over the telephone (WTP £642.13) or in an individual discussion (WTP £594.32). Members of this group were averse to receiving information via email (WTP −£1350.51). This group preferred receiving dietary advice at their GP surgery and did not want to receive it at a community centre. The older participants were the more likely to fall into the “one-to-one” category.

The final category (*n* = 39, 22%) was labelled the “person-centred” communicators, who valued direct in-person dietary advice. They value being able to receive healthy lifestyle advice face-to-face, whether this was via individual discussion (WTP £358.79) or group discussion (WTP £290.62). Receiving information via the indirect methods, telephone calls and emails, was strongly disliked by this group. Despite having strong opinions about how they would prefer to receive information, the “person-centred” group did not have strong preferences about where they wanted to receive information. Members of this group were more likely to have high self-reported risk and were more likely to be female.

Other demographic factors including education, years of education, ethnicity, site of surgery, smoking status, recruitment site and whether the patient had a stoma did not predict membership of the groups.

Across all groups, there was a universal preference for information to be provided by a specialist bowel cancer nurse. Participants were generally against or indifferent to receiving advice from a dietician. All groups preferred not to receive healthy lifestyle advice from a general practice nurse.

## Discussion

The use of a BWDCE has highlighted some novel findings regarding preferences for dietary advice after treatment for people who have survived colorectal cancer. Exploration of heterogeneity within the cohort has identified different preferences for lifestyle advice in groups who have similar characteristics. The identification of sub-groups within the total colorectal cancer survivorship population adds to the evidence base. This experiment has shown people who require dietary intervention and who regard themselves as most at risk would prefer face-to-face advice with a specialist bowel cancer nurse at a hospital (“person-centred” group). However, younger males who indicated they were adhering to current guidelines stated they would prefer information in their own home using email (“technophiles”). Older people were more likely to favour telephone contact or face-to-face consultations at their GP surgery (“one-to-one” group). The data provide evidence that for healthy lifestyle specifically relating to dietary interventions, there is not one style that is suitable for all people.

The use of the BWDCE method allowed twice the amount of data to be captured compared to a traditional DCE, leading to increased statistical precision in the parameter estimates. At an aggregate level, information provided in a group discussion or via email was disliked by participants, albeit there was heterogeneity between groups. The inconsistencies apparent between participants’ choices for the best and worst interventions in a choice set provide an indication that preference heterogeneity may exist with regard to the provision of dietary advice.

Previous attempts to design and evaluate intervention services for people surviving cancer have relied on a universal approach. However, to date, such approaches have resulted in only modest changes to high-risk health behaviours particularly in people with colorectal cancer [29]. As a result, the effectiveness and cost-effectiveness of such interventions in preventing recurrence of cancer appears limited.

One potential cause of this limited impact may be the presence of heterogeneity with regard to people’s preferences for receiving dietary interventions. This study suggested that there may be a range of categories of participants with different preferences for how they receive information. Participants’ self-reported adherence to healthy lifestyle recommendations was one of the key predictors of membership in these categories. Those with adherence to fewer recommendations had a higher risk associated with future malignancies and were a category of participants who prefer information to be delivered in face-to-face individual or group discussions. On the other hand, people who followed more of the healthy lifestyle recommendations were more likely to belong to a category of people preferring information via telephone calls, emails and individual discussions.

Following on from the results, it would seem that the best approach to provide dietary advice may therefore be to tailor information to different groups of people based on their preferences. Groups who adhered to fewer healthy lifestyle recommendations exhibiting more risky behaviours potentially have a greater risk of further malignancy. Following a greater number of healthy lifestyle recommendations has been associated with a lower hazard ratio of dying from cancer, circulatory disease or respiratory disease [30]. This group would therefore be most likely to benefit from healthy lifestyle and dietary advice. However, this study suggested that their preference for face-to-face discussion is not being met by the current trend for telephone-based information. Failing to engage this specific group in behavioural change may present a missed opportunity.

Furthermore, the preference of individuals with a lower risk for an intervention via telephone calls or emails may present a more cost-effective way of delivering an intervention. These modes of information provision are likely to be less costly to healthcare providers and target people who are less likely to benefit from behavioural change. Personalisation of healthy lifestyle advice may therefore facilitate the more efficient use of healthcare resources. In order to fully evaluate the most cost-effective manner of providing healthy lifestyle advice, an economic evaluation building on a clinical trial would be required. In this way, the potential benefits gained from personalising lifestyle information in terms of reducing cancer recurrence could be systematically compared to the additional costs which may be incurred.

There were some limitations to this study. Firstly, there were a significant number of participants in this study who had no formal education. This may mean that the sample used in this study was not representative of the wider patient population and that the conclusions of this paper should be interpreted with care. However, in the latent class analysis, educational level was not found to be a predictor of how patients preferred to receive information.

Furthermore, the WTP values calculated from analysis of best and worst choices separately differ significantly. If best choices are the opposite of worst choices, as is assumed in the combined models, these values should be equal. This problem has been commonly observed in previous best-worst scaling experiments [18]. Some of this effect may be due to heterogeneity in participants’ best and worst choices. For example, the contradiction in WTP values for telephone calls and group discussions can be explained by the assumption that some groups of people prefer these modes whilst others dislike them. It has also been suggested that participants in best-worst discrete choice experiments may use more simplistic methods when making worst choices. For example, participants may always choose profiles with a given level of an attribute as worst. This may be the case with the email level, which was chosen as worst by almost all participants other than younger males. This may indicate that the participants were not fully evaluating each profile. DCEs are grounded on theories of rational consumer choice. As with any applied choice experiment, it is difficult to ascertain the extent to which participant responses adhere to assumptions of economic theory. Systematic differences in how participants chose best and worst profiles cannot be entirely ruled out in attempts to explain apparent inconsistencies in WTP values.

## Conclusions

The key findings of this preference-based study were that different preferences are expressed by people who have survived colorectal cancer regarding the delivery of healthy eating intervention. Factors to take into consideration are age, self-reported lifestyle behaviours and gender. At an aggregate level, preferences were for a dietary intervention delivered by a bowel specialist nurse, locally and by an individual discussion either face-to-face or on the telephone. However, the additional data provided by the best-worst methodology has allowed categories of people to be highlighted that would be more likely to prefer alternative delivery of dietary information either by email or group sessions. The key to determining the likelihood of preferences was the individual’s age, gender and self-assessed risk based on healthy lifestyle recommendations for the prevention of cancer [20]. The three groups, “technophiles”, “one to one” and “person-centred” communicators, are characterised by preferences based on mode of delivering information, place and professional, and this is influenced by their overall risk, age and gender. This information can be used in future when designing interventions to ensure the right individuals are targeted by the right approach since clearly “one size does not fit all”.

BMI, body mass index; BWDCE, best-worst scaling discrete choice experiments; DCEs, discrete choice experiments; GP, general practitioner; SD, standard deviation; WTP, willingness to pay.

## Electronic supplementary material

ESM 1 (DOCX 18 kb)

## References

[1] Ferlay J (2010). Estimates of worldwide burden of cancer in 2008: GLOBOCAN 2008. Int J Cancer.

[2] Maddams J (2009). Cancer prevalence in the United Kingdom: estimates for 2008. Br J Cancer.

[3] Maddams J, Utley M, Moller H (2012). Projections of cancer prevalence in the United Kingdom, 2010-2040. Br J Cancer.

[4] Khan NF (2010). Consulting and prescribing behaviour for anxiety and depression in long-term survivors of cancer in the UK. Eur J Cancer.

[5] Maddams J, Utley M, Moller H (2011). Levels of acute health service use among cancer survivors in the United Kingdom. Eur J Cancer.

[6] Heins MJ (2013). For which health problems do cancer survivors visit their general practitioner?. Eur J Cancer.

[7] Nobbs HM (2016). Do dietary patterns in older age influence the development of cancer and cardiovascular disease: a longitudinal study of ageing. Clin Nutr.

[8] Evans HS (2002). The risk of subsequent primary cancers after colorectal cancer in southeast England. Gut.

[9] Yabroff KR (2004). Burden of illness in cancer survivors: findings from a population-based national sample. J Natl Cancer Inst.

[10] Demark-Wahnefried W (2005). Riding the crest of the teachable moment: promoting long-term health after the diagnosis of cancer. J Clin Oncol.

[11] Morey MC (2009). Effects of home-based diet and exercise on functional outcomes among older, overweight long-term cancer survivors: RENEW: a randomized controlled trial. JAMA.

[12] Miller P (2008). Dietary supplement use among elderly, long-term cancer survivors. J Cancer Surviv.

[13] Hawkes AL (2013). Effects of a telephone-delivered multiple health behavior change intervention (CanChange) on health and behavioral outcomes in survivors of colorectal cancer: a randomized controlled trial. J Clin Oncol.

[14] Anderson AS, et al. The impact of a bodyweight and physical activity intervention (BeWEL) initiated through a national colorectal cancer screening programme: randomised controlled trial. BMJ. 2014;348 10.1136/bmj.g1823 PMC3945930 24609919

[15] Finocchiaro C (2016). Effect of specific educational program on dietary change and weight loss in breast-cancer survivors. Clin Nutr.

[16] Kushi LDC, McCullough M, Rock CR, Demark-Wahnefried W, Bandera EV, Gapstur E, Patel AV, Andrews K, Gansler T, The American Cancer Society 2010 Nutrition and Physical Activity Guidelines Advisory Committee (2012). American Cancer Society Guidelines on nutrition and physical activity for cancer prevention: reducing the risk of cancer with healthy food choices and physical activity. CA Cancer J Clin.

[17] Craig P (2008). Developing and evaluating complex interventions: the new Medical Research Council guidance. Br Med J.

[18] Clark MD (2014). Discrete choice experiments in health economics: a review of the literature. PharmacoEconomics.

[19] Lancsar E (2013). Best worst discrete choice experiments in health: methods and an application. Soc Sci Med.

[20] Flynn T. Valuing citizen and patient preferences in health: recent developments in three types of best–worst scaling. Expert Rev Pharmacoecon Outcomes Res. 2010:10(3). 10.1586/erp.10.29 20545591

[21] Flynn TN (2007). Best–worst scaling: what it can do for health care research and how to do it. J Health Econ.

[22] Burden ST (2016). An exploration of food and the lived experience of individuals after treatment for colorectal cancer using a phenomenological approach. J Hum Nutr Diet.

[23] Burden S, Gibson DJ, Todd C, Gratton EK, Pilling M, Lal S. Dietary interventions for adult cancer survivors. Cochrane Database of Systematic Reviews Issue 9. Art. No.: CD011287, 2014(9). doi: 10.1002/14651858.CD011287. 10.1002/14651858.CD011287.pub2 PMC6872979 31755089

[24] ChoiceMetrics, NGene. 2014.

[25] StataCorp, Stata 14. 2016.

[26] Statistical Innovations, Latent Gold Choice 5.1. 2016.

[27] Burke P (2010). The scale-adjusted latent class model: application to museum visitation. Tour Anal.

[28] Watson V, Becker F, de Bekker-Grob E. Discrete choice experiment response rates: a meta-analysis. Health Econ. 2016. doi:10.1002/hec.3354. 10.1002/hec.3354 27122445

[29] Rock CL, Demark-Wahnefried W (2002). Nutrition and survival after the diagnosis of breast cancer: a review of the evidence. J Clin Oncol.

[30] Vergnaud A-C (2013). Adherence to the World Cancer Research Fund/American Institute for Cancer Research guidelines and risk of death in Europe: results from the European Prospective Investigation into Nutrition and Cancer cohort study. Am J Clin Nutr.

