# Supplementary material for: What are colorectal cancer survivors’ preferences for dietary advice? A best-worst discrete choice experiment
Source: J Cancer Surviv. 2017 Apr 20;11(6):782–90. doi: 10.1007/s11764-017-0615-2 (PMC5671542; doi:10.1007/s11764-017-0615-2)
Supplement: Supplementary file 1 — (DOCX 18 kb) [file 11764_2017_615_MOESM1_ESM.docx]

Supplementary material: Technical details

**Sample Size Calculation**

A variety of factors, such as the efficiency of the design and level of heterogeneity of responses, impact the necessary sample size for a DCE and as such this is difficult to calculate [[12](#_ENREF_12)]. Here we use an ad hoc method proposed by Johnson and Orme:

$$\frac{nta}{c} \geq500$$

“where n is the number of respondents, t is the number of tasks, a is number of alternatives per task (not including the none alternative), and c is the number of analysis cells”. In this study, t=12, a=4 and c=16 (in order to calculate two-way interactions) and by rearranging we arrive at a minimum required sample size of 167. As this calculation is intended for standard DCEs and more data will be obtained from the best-worst approach used in this study, this sample size was deemed sufficient to obtain good estimates of the coefficients of interest.

**Data Analysis**

Conditional Logistic Regression of Best and Worst Choices

In order to identify the attributes and levels which contributed to a scenario being chosen as the best or worst in a given choice set, two separate conditional logistic regressions were conducted. Conditional logistic regression models have the advantage of controlling for the fact that different sets of observations result from the answers of different individuals. For the regression of the best choices, the outcome variable was coded as a 1 for the profile chosen as best and 0 for the other profiles. The independent variables comprised the attributes and levels appearing in the profiles.

For the worst choice conditional logistic regression, the outcome variable was coded as 1 for the profile chosen as worst and 0 for the other profiles. The independent variables comprised the attributes and levels appearing in the profiles.

This analysis aimed to identify differences in the choice of the best and worst profiles.

Sequential Best-Worst Logistic Regression

A sequential best-worst logistic regression model was used to combine the best and worst choices at an aggregate level. In this model, the effect of attributes and levels occurring in profiles chosen as worst is inversed (multiplied by -1). As such, attributes and levels occurring in profiles that are chosen as worst are treated as reducing the probability that a profile in the combined analysis is chosen as best. This involves the assumption that worst is the opposite of best. This assumption is investigated in the separate best and worst analysis.

**Scale adjusted latent class sequential best-worst logistic regression**

This model builds on the sequential best-worst logistic regression by incorporating preference heterogeneity. In this model, groups of individuals with similar preferences are identified. Further analysis is used to identify whether any demographic information predicts membership of these latent classes. This allows the labelling of these groups in a more interpretable way.

Identifying the number of latent classes to include is a matter of interpretation by the researcher. In a similar way that adding additional explanatory variables to a linear model always improves the R^2^ value, adding additional latent classes always improves the log-likelihood (a measure of model fit) of a latent class model. In order to counter this, different measure are used to assess the appropriateness of a latent class model. The main measures used are the Akaike Information Criterion (AIC) and Bayesian Information Criterion (BIC). These models represent the amount of information which is not explained by the model meaning that lower number are better. However, both to varying degrees punish a model for adding more variables.

However, in the models in this study, even these criteria continuously improved as more classes were added. Three latent classes and three scale classes were chosen for the model as beyond this number, adding additional classes provided only minimal benefit in terms of the explanatory power of the model.

In addition to preference heterogeneity, this model also allows for scale heterogeneity. In a logistic regression, the magnitude of the coefficients is a function of the error involved in the elicitation of those coefficients. In other words, the consistency of individuals in expressing their preferences can impact on the size of the coefficients. This is a potentially important consideration when preference heterogeneity is analysed. If different groups of individuals have different choice consistencies, the coefficients of the attributes and levels can falsely appear to be different when in fact they are the same but contain different levels error. This is particularly important for latent class models as individuals can be placed into the wrong latent class based on the appearance of their preferences.

To account for differences in scale, a scale-adjusted latent class model is used. A similar method is used to that for adding additional preference classes. The model identified in this study included three scale classes. However, in order to avoid confusion, we only present one of these classes (the largest) with willingness to pay estimates. As willingness to pay estimates are ratios, they are not impacted by scale effects and can therefore be compared across the latent classes.

**Willingness to Pay**

In this best worst discreet choice experiment, a cost attribute was included in order to calculate the willingness to pay for an attribute or level. This can be done by calculating the marginal rate of substitution between an attribute and the cost. More simply, this represents how much the cost of a profile containing a given attribute or level could rise before it would no longer be chosen. This value represents how much the participants (at an aggregate level) were willing to pay for an attribute or level.

These willingness to pay values are easier to interpret than the coefficients of the discrete choice experiment, which as previously mentioned may be influenced by choice consistency. However, it should be noted that as effects coding has been used in this study for the categorical variables, the willingness to pay represents how much the participants would be willing to pay for a scenario containing a given attribute or level relative to a dietary advice programme of “average” value.
